# Only Tumors Angiographically Identified as Hypervascular Exhibit Lower Intraoperative Blood Loss Upon Selective Preoperative Embolization of Spinal Metastases: Systematic Review and Meta-Analysis

**DOI:** 10.3389/fonc.2020.597476

**Published:** 2021-01-19

**Authors:** Yining Gong, Changming Wang, Hua Liu, Xiaoguang Liu, Liang Jiang

**Affiliations:** ^1^ Department of Orthopaedics, Peking University Third Hospital, Beijing, China; ^2^ Health Science Center, Peking University, Beijing, China; ^3^ Department of Interventional Radiology and Vascular Surgery, Peking University Third Hospital, Beijing, China

**Keywords:** hypervascular tumor, angiography, spinal metastases, intraoperative blood loss, preoperative embolization, systematic review and meta-analysis

## Abstract

**Background:**

The role of preoperative embolization (PE) in reducing intraoperative blood loss (IBL) during surgical treatment of spinal metastases remains controversial.

**Methods:**

A systematic search was conducted for retrospective studies and randomized controlled trials (RCTs) comparing the IBL between an embolization group (EG) and non-embolization group (NEG) for spinal metastases. IBL data of both groups were synthesized and analyzed for all tumor types, hypervascular tumor types, and non-hypervascular tumor types.

**Results:**

In total, 839 patients in 11 studies (one RCT and 10 retrospective studies) were included in the analysis. For all tumor types, the average IBL did not differ significantly between the EG and NEG in the RCT (*P* = 0.270), and there was no significant difference between the two groups in the retrospective studies (*P* = 0.05, standardized mean difference [SMD] = −0.51, 95% confidence interval [CI]: −1.03 to 0.00). For hypervascular tumors determined as such by consensus (n = 542), there was no significant difference between the two groups (*P* = 0.52, SMD = −0.25, 95% CI: −1.01 to 0.52). For those determined as such using angiographic evidence, the IBL was significantly lower in the EG than in the NEG group, in the RCT (*P* = 0.041) and in the retrospective studies (*P* = 0.004, SMD = −0.93, 95% CI: −1.55 to −.30). For IBL of non-hypervascular tumor types, both the retrospective study (*P* = 0.215) and RCT (*P* = 0.947) demonstrated no statistically significant differences in IBL between the groups.

**Conclusions:**

Only tumors angiographically identified as hypervascular exhibited lower IBL upon PE in this study. Further exploration of non-invasive methods to identify the vascularity of tumors is warranted.

## Introduction

Cancers of various origins metastasize frequently to bone, and the spine is the most common location of bone metastases (1, 2). To relieve pain, improve neurological function, and maintain spinal stability and local tumor control, surgical intervention is necessary for certain spinal metastases (1, 3, 4). However, such surgery carries a high risk of extreme intraoperative blood loss (IBL), especially for highly vascularized tumors (5). Massive bleeding may obscure the surgical field and lengthen or even interrupt the operation, resulting in higher mortality and morbidity. Data indicate that a large proportion of spinal metastases are hypervascular, especially those from renal cell cancer (RCC), hepatocellular cancer (HCC), and thyroid cancer (6–8).

Selective embolization in highly vascular bone tumors was first described by Feldman et al. (9). Usually, it is performed *via* an angiogram catheter, and preoperative embolization (PE) has been proven to be safe (1). PE is designed to provide a clean view of the surgical field, which could improve surgical efficiency and minimise complications. The current treatment paradigm utilizes PE mainly for hypervascular tumors such as metastases from RCC, HCC, and thyroid cancer (8). Several studies indicated that PE reduced IBL (5, 10–14), while others revealed no statistically significant difference in IBL between patients undergoing and those not undergoing PE (4, 8, 15–17).

Thus, the role of PE for spinal metastases remains controversial. A meta-analysis, conducted by Luksanapruksa et al. (18), included five studies conducted between 1993 and 2015. They concluded that PE could be useful in reducing IBL in surgery for spinal metastases of both renal cell carcinoma and mixed tumors. Here, we aimed to verify whether all hypervascular tumors can benefit from PE.

## Methods

### Search Strategy

The study was conducted in accordance with the Preferred Reporting Items for Systematic Reviews and Meta-Analyses statement (19). We registered our study protocol prospectively using PROSPERO (CRD42020159660). We conducted a systematic search of published studies using PubMed, Web of Science, EMBASE, and the Cochrane Library. Additionally, we performed a manual search using the reference lists of eligible studies and reviewed articles to identify further potentially eligible studies. Search dates were between November 24, 2019 and 28, 2019. Only English-language literature published between 1990 and 2019 was considered. We repeated the search just before the final analyses to identify and retrieve any further studies that had been published in the interim. Unpublished studies were not searched.

We used the following keywords to conduct our search, using wildcards to include spelling variants and plurals: (“embolization” OR “embolism”) AND (“spin* metastases” OR “spinal metastatic diseases” OR “metastatic spinal tumor” OR “spinal bone metastases”). Electronic searches were performed independently by two reviewers.

### Selection Criteria

All accessible studies were screened for eligibility. Inclusion criteria were as follows: (1) patients had a confirmed diagnosis of spinal metastases; (2) surgical treatment was applied; (3) PE was conducted; (4) an embolization group (EG) was compared with a non-embolization group (NEG); and (5) the mean and standard deviation (or the median and range) IBL were recorded. Exclusion criteria were as follows: (1) the cohort included patients with primary spinal tumors that could not be separated from those with spinal metastases; (2) IBL values of the EG and NEG could not be obtained separately; (3) either or both groups contained <10 patients; and (4) data was reused by the same research group. Both randomized controlled trials (RCTs) and retrospective studies were included; however, non-clinical research articles (*e.g.* case reports, letters, reviews, conference papers, animal studies, and basic medical research) were excluded. Study selection was performed independently by two reviewers.

### Data Extraction

After eligible studies were selected, two reviewers performed data extraction independently. Included studies were reviewed to extract (1) study characteristics, (2) patient demographic data, (3) tumor characteristics, (4) embolization techniques, (5) surgical procedure details, (6) IBL, and (7) complications. Data were recorded in an Excel spreadsheet (Microsoft Corp., Redmond, WA, USA). Disagreements were resolved by discussion and the involvement of a third reviewer.

### Assessment of Study Quality

Study quality was assessed at the study level; the Cochrane risk-of-bias tool was used for RCTs, and the Newcastle-Ottawa Scale for retrospective studies. The former was used to assess randomization, blinding, outcome assessment, and methods of analysis to classify RCTs as posing a low, unclear, or high risk of bias. The latter was used to assess the following: selection (representativeness of the exposed cohort; selection of the non-exposed cohort; ascertainment of exposure to implants; and demonstration that outcome of interest was not present at start of study), comparability (comparability of cohorts on the basis of the design or analysis) and outcome (assessment of outcome; was follow up long enough for outcomes to occur; adequacy of follow up of cohorts). Disagreements between the two independent reviewers were resolved by discussion.

### Data Analysis

Data were pooled and analyzed using Review Manager (RevMan, version 5.3; The Nordic Cochrane Centre, The Cochrane Collaboration, Copenhagen, Denmark). The frequency distribution and the means ± standard deviations (SDs) were used to describe and summarize the items. The means and SDs, where unavailable, were estimated using the medians and ranges, *via* the method proposed by Hozo et al. (20). The standardized mean difference (SMD) functioned as a measure of the effect size during IBL analyses. A 95% confidence interval (CI) was also employed.

Heterogeneity was examined using inconsistency (I) statistics. I^2^ >50% was considered to indicate substantial heterogeneity, and a random-effects model was used for analyses using such data. When I^2^ ≤50%, a fixed-effects model was tested. In the presence of substantial heterogeneity, we performed sensitivity analyses, omitting one study at a time to examine the effect on the results. A *P*-value <0.05 was regarded statistically significant. Publication bias was examined using funnel plots. Symmetrical funnel plots located within the 95% confidence region demonstrated no publication bias.

## Results

### Literature Search and Assessment of Study Quality

**Figure 1:**
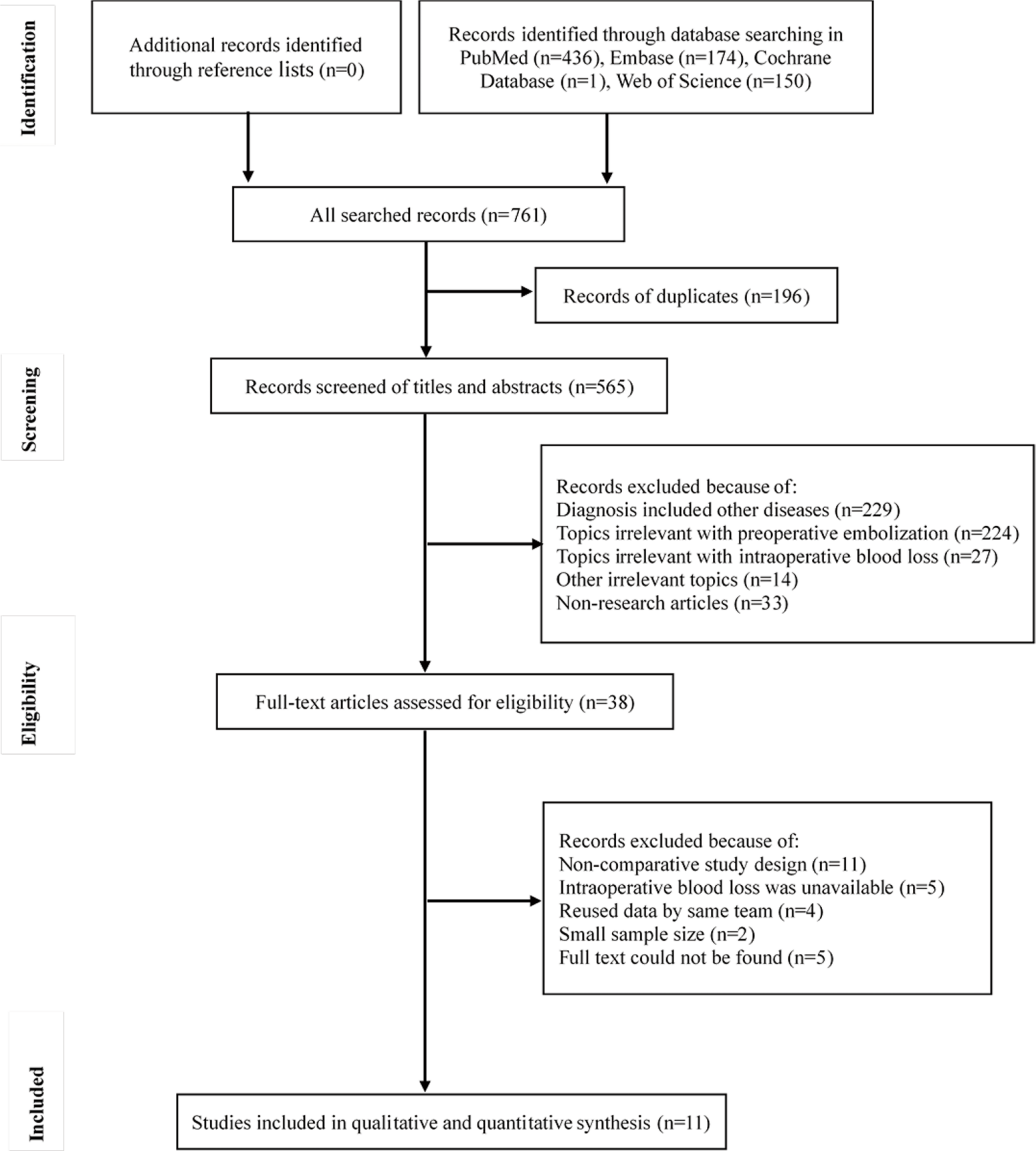
The flow of patients in each phase.

In total, 761 studies were identified initially. After removing duplicates, screening titles and abstracts, and assessing full text, 11 studies were included in the analyses (4, 5, 8, 10, 12–16, 21, 22). The flow of information through the different phases is displayed in **Figure 1**. One RCT and 10 retrospective studies were identified. The RCT exhibited a low risk of bias in terms of random sequence generation, allocation concealment, blinding of participants and personnel, blinding of outcome assessment, incomplete outcome data, and selective reporting. The Newcastle-Ottawa Scale scores demonstrated that the retrospective studies were of high quality (**Table 1**).

**Table 1:** Study characteristics and demographics.

Study	Country	NOS	Study design	No. of patients	Age	Sex (M/F)	Tumor histology	Embolization material	Extent of embolization	Interval between embolization and surgery (h)
Yoo et al. (16)	Korea	9	Retrospective	79	57.6 ± 13.5	50/29	Mixed (non-hypervascular)	NA	NA	<48
Zaborovskii et al. (4)	Russia	9	Retrospective	54	61.6 ± 3.3	41/13	RCC	GS	NA	<48
Hong et al. (14)	Korea	9	Retrospective	52	59.7 ± 12.3	37/15	Mixed	PVA, GS	Complete or near-complete (72.2%), partial (27.8%)	<48
Tan et al. (8)	Singapore	7	Retrospective	209	NA	NA	Mixed	GS, PVA, coils	Total (61%), subtotal (29%), partial (10%)	<48
Kumar et al. (21)	Singapore	7	Retrospective	218	60.0 ± 12.4	110/108	Mixed	GS, PVA, coils	Total (53%), subtotal (36%), partial (11%)	<48
Clausen et al. (15)	Denmark	ROB	RCT	45	63.5 ± 8.1	28/17	Mixed	PVA, GS, coils	Grade 3 (82.6%), grade 2 (13.0%), grade 1 (4.3%)	<48
Kato et al. (13)	Japan	9	Retrospective	46	61.0 ± 15.5	28/18	Mixed	PVA, GS, coils	Complete (30.4%), partial (69.6%)	<72
Wirbel et al. (5)	Germany	7	Retrospective	41	NA	NA	Mixed	Coils, Contour Emboli particles	Complete (95%)	<24
Manke et al. (12)	Germany	7	Retrospective	32 (lesions)	63.3 ± 9.1	21/11	RCC	PVA	Complete (50%), partial (50%)	<48
Hess et al. (10)	Germany	8	Retrospective	34	NA	NA	RCC, thyroid cancer and adenocarcinoma	Coils, Contour Emboli particles	Complete (88.2%)	<48
Olerud et al. (22)	Sweden	7	Retrospective	29 (operations)	63.4 ± 9.3	15/14	RCC	PVA, GS	NA	<24

Age is indicated as the mean ± standard deviations. NOS, Newcastle-Ottawa Scale; ROB, Risk of Bias; RCT, randomized controlled trial; NA, not available; M/F, male/female; RCC, renal cell cancer; GS, gelatin sponge; PVA, polyvinyl alcohol particles.

### Study Characteristics and Demographics

Altogether, 839 patients in 11 studies were included in the analysis. The studies were conducted in seven countries, all from Europe or Asia. The publication years ranged from 1993 to 2019, and data-collection years ranged from 1981 to 2016. The average age of patients ranged from 57.6 to 63.5 years. The male-to-female ratio was approximately 1.5. Most studies included mixed tumors (determined *via* histology); four (4, 10, 12, 22) focused on hypervascular tumors, especially RCC. In contrast, Yoo et al. (16) performed research particularly on non-hypervascular tumors. Rather than using traditional histology, the authors of four studies determined hypervascularity using pre-embolization angiography (4, 5, 12, 15). The common materials used for embolization were gelatin sponge, polyvinyl alcohol particles, and metallic coils. The definition of the extent of embolization varied between studies. In 10/11 studies, the time interval between embolization and surgery was <48 h; in two of these, it was <24 h (**Table 1**). In terms of the operations, both anterior and posterior approaches were applied. Most patients underwent vertebrectomies or decompression surgeries. Usually, IBL was estimated by the amount of suction drainage, the amount of blood soaked by gauze, or hemoglobin levels.

### IBL of All Tumor Types

**Figure 2:**
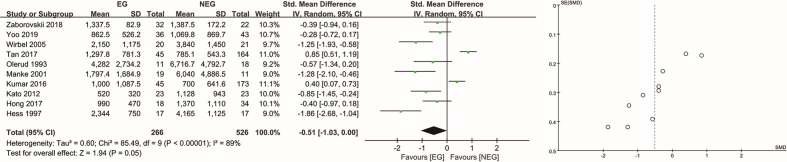
Analysis of intraoperative blood loss in all tumor types. EG, embolization group; NEG, non-embolization group; SD, standard deviation; CI, confidence interval; IV, inverse variance; SE, standard error; SMD, standardized mean difference.

In total, the IBL of 837 patients was analyzed, including 289 patients in the EG and 548 in the NEG. Two patients in the study by Manke et al. (12) were excluded, one on whom no operation was performed and one for whom IBL data was missing. In the RCT (15), the average IBL did not differ significantly (*P* = 0.270) between the EG [618 ml (SD, 282 ml)] and NEG [735 ml (SD, 415 ml)]. In the retrospective studies (4, 5, 8, 10, 12–14, 16, 21, 22), the average IBL in the EG and NEG was 1,395 ml [SD, 1,252 ml] and 1,402 ml [SD, 1,979 ml], respectively. There was no significant difference between the two groups (*P* = 0.05), and the SMD was −0.51 (95% CI: −1.03 to 0.00, I^2^ = 89%, random-effects) (**Figure 2**). Upon sensitivity analysis, the difference was significant after removing the study by Tan et al. (8) (*P* = 0.005, SMD = −0.67, 95% CI: −1.13 to −0.20, I^2^ = 83%, random-effects) or Kumar et al. (21) (*P* = 0.03, SMD = −0.63, 95% CI: −1.22 to −0.05, I^2^ = 89%, random-effects), although there was no significant difference when omitting any of the other studies (see **Supplementary Material**).

### IBL of Hypervascular Tumor Types

Of the hypervascular tumors, 542 were recognized as such by consensus (RCC, HCC, thyroid cancer) and 159 using angiographic evidence.

**Figure 3:**
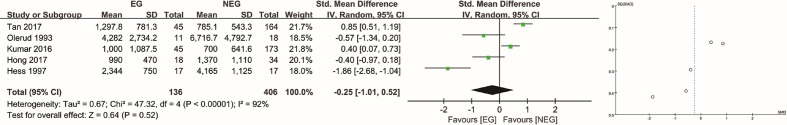
Analysis of intraoperative blood loss in hypervascular tumors determined as such by consensus. EG, embolization group; NEG, non-embolization group; SD, standard deviation; CI, confidence interval; IV, inverse variance; SE, standard error; SMD, standardized mean difference.

For patients with hypervascular tumors determined as such by consensus (8, 10, 14, 21, 22), the average IBL in the EG and NEG was 1531 ml [SD, 1,443 ml] and 1,202 ml [SD, 1,819 ml], respectively. There was no significant difference between the two groups (*P* = 0.52), and the SMD was −0.25 (95% CI: −1.01 to 0.52, I^2^ = 92%, random-effects) (**Figure 3**). Upon sensitivity analyses, the difference was significant (*P* = 0.04, SMD = −0.91, 95% CI: −1.77 to −0.05, I^2^ = 77%, random-effects) only after removing the studies by both Tan et al. (8) and Kumar et al. (21). There was no significant difference when omitting any other studies (see **Supplementary Materials**).

**Figure 4:**
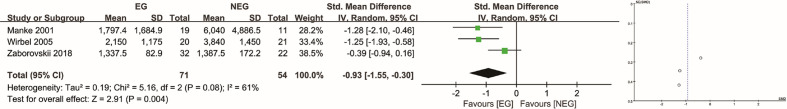
Analysis of intraoperative blood loss in hypervascular tumors determined as such using angiographic evidence. EG, embolization group; NEG, non-embolization group; SD, standard deviation; CI, confidence interval; IV, inverse variance; SE, standard error; SMD, standardized mean difference.

In the RCT (15), for patients with hypervascular tumors determined as such using angiographic evidence, the analysis revealed a significantly (*P* = 0.041) lower IBL in the EG compared to the NEG (645 ml [SD, 289 ml] *vs*. 902 ml [SD, 416 ml]). The tumor types analyzed in the RCT were renal, breast, lung, head and neck, and colon tumors, as well as melanomas. In the retrospective studies in which hypervascular tumors were determined as such using angiographic evidence (4, 5, 12), the tumor types included renal, thyroid, and breast tumors, as well as plasmacytomas. In these, the average IBL in the EG and NEG was 1,689 ml [SD, 1,108 ml] and 3,289 ml [SD, 3,042 ml], respectively. There was a significant difference between the two groups (*P* = 0.004), and the SMD was −0.93 (95% CI: −1.55 to −0.30, I^2^ = 61%, random-effects) (**Figure 4**) (for sensitivity analyses, see **Supplementary Materials**).

### IBL of Non-Hypervascular Tumor Types

Two studies reported the outcomes of IBL of non-hypervascular tumor types (15, 16). Yoo et al. (16), in a retrospective study, demonstrated that there was no significant difference in IBL between the EG and NEG (863 ml [SD, 526 ml] *vs*. 1,070 ml [SD, 870 ml], *P* = 0.215). However, HCCs were also included in their analysis, which are considered hypervascular tumors by consensus. In the RCT by Clausen et al. (15), analysis of the non-hypervascular subgroup revealed that IBL in the EG did not differ significantly from that in the NEG (437 ml [SD, 152 ml] *vs*. 445 ml [SD, 209 ml], *P* = 0.947).

### Assessment of Publication Bias

The outcome of the assessment was summarised using funnel plots; there was no evidence of obvious publication bias.

## Discussion

In the present study, we compared the IBL during surgery for spinal metastases in patients undergoing or not undergoing PE and drew a different conclusion from the authors of a previous meta-analysis (18). A reduction in IBL after embolization was only observed during surgery for spinal metastases of hypervascular tumors determined as such using angiographic evidence. There were no statistically significant differences in IBL when all histological tumor types were pooled together or even for hypervascular tumors determined as such by consensus (RCC, HCC, thyroid cancer).

In the combined analysis of all tumor types, the average IBL did not differ statistically significantly between the EG and NEG in both the RCT and the retrospective studies. Therefore, we do not think it is reasonable to perform PE for all spinal metastases. There was a significant difference between the two groups in the retrospective analyses after removing the studies by Tan et al. (8) or Kumar et al. (21) during sensitivity analyses. However, massive blood loss was observed more frequently in tumors regarded traditionally as hypervascular, and embolization was more frequently performed among such tumors (14, 23). As hypervascular tumors are overrepresented in the studies analyzed here and in the previous meta-analyses, the results are not generalizable to all tumor types.

For tumors determined as hypervascular upon angiography, PE seemed to be effective in reducing IBL, with statistical and clinical support from both the results of the RCT and the synthesis of the retrospective studies. However, for hypervascular tumors determined as such by consensus, there was no difference between the EG and NEG. According to Tan et al. (8), only approximately 67% of RCCs and 60% of HCCs appear hypervascular upon angiography. It is doubtful when to make the decision to perform PE based on tumor pathology. Meanwhile it may explain in part why different studies’ conclusions differ on this topic. In the studies by Tan et al. (8) and Kumar et al. (21), IBL was lower in the NEG than in the EG. It may be partly due to differences in characteristics before treatment, such as the uneven distribution of histological tumor types, metastasis size, the extent of the tumors, corporeity vulnerable to bleeding, the technical complexity and time of the surgery, and the completeness of PE (6, 8, 21). However, some of these factors are difficult to compare, especially in retrospective studies.

For the two studies in which IBL of surgery for spinal metastases of non-hypervascular tumor types was reported, there was no difference in IBL between the EG and NEG. However, although massive bleeding was more frequent among hypervascular tumor patients, it was also observed in several patients with non-hypervascular tumors (14). Hypervascularity has also been observed in typically non-hypervascular tumors; *e.g.* in one study, 43% of lung cancer spinal metastases had a hypervascular angiographic appearance (8). Other studies also demonstrated this phenomenon in breast, lung, head-and-neck, colon, prostate, and hematological malignancies, as well as melanomas (7, 15). This highlights the need to expand our perception on which spinal malignancies may be hypervascular and may benefit from PE.

Only patients with hypervascular tumors determined as such using angiographic evidence derived definite benefits from PE in our analysis. In the study by Zaborovskii et al. (4), tumor vascularity was evaluated by an interventional radiologist and classified into four grades (not hypervascular, mild, moderate, and severe) according to the severity of tumor blush and venous drainage. Clausen et al. (15) graded the vascularity of the metastases, by visual evaluation of the intensity of tumor blush, as: no hypervascularity (equal to or less than adjacent vertebrae without tumor involvement), moderate hypervascularity, and pronounced hypervascularity. Lesions were assigned a vascularity grade (1–3) according to tumor blush in the study by Meng et al. (24). Their criteria were as follows: grade 1 (hypovascular) for tumors with a vascularity or weak tumor blush equal to that of adjacent vertebral bodies without tumor involvement; grade 2 (moderate) for tumors with a moderate tumor blush greater than that of the adjacent vertebral bodies; and grade 3 (hypervascular) for tumors with a substantial tumor blush with arteriovenous shunting. However, there are no widely accepted criteria for the classification of hypervascularity. Therefore, a well-organized definition of hypervascularity would improve the replicability of future research in the field.

Spinal cord infarction and deterioration of neurological status were major complications of selective embolization in this study. Because angiographic vascularity is not always consistent with histological tumor type, development of non-invasive methods to identify hypervascular tumors can reduce unnecessary costs and complications, and avoid the exclusion of unexpected hypervascular tumors. Meng et al. (24), by analyzing its correlation with angiography, demonstrated that dynamic contrast-enhanced magnetic resonance imaging is an accurate technique for the assessment of spinal-tumor vascularity. They modified the criteria developed by Thiex et al. (7) to determine tumor hypervascularity using magnetic resonance imaging. Their criteria were as follows: grade 1 (hypovascular), no or mild gadolinium enhancement compared with the blush of adjacent, uninvolved vertebral bodies; grade 2 (moderate), moderate gadolinium enhancement; and grade 3 (hypervascular), avid gadolinium enhancement, and evidence of intra- or peritumoral flow voids or intratumoral hemorrhage. This technique may serve as a screening method before angiography and embolization. However, more evidence is needed to verify this new application.

This study has some limitations. First, the amount of hemorrhage relies on a combination of various parameters, such as the degree of vascularity, the size and extent of the tumors, the complexity and duration of the surgery, the surgical approach, and the extent of embolization. Although studies with small sample sizes were excluded, several parameters remained difficult to compare in the retrospective studies. Therefore, prospective studies should be conducted with these parameters in mind. Second, the measurement details of IBL were difficult to distinguish among studies, which could introduce bias, especially when the difference between the EG and NEG was small. Finally, there were high levels of heterogeneity in this analysis. Therefore, we performed sensitivity analyses and partly attributed the differences in the outcomes to the inconsistency between determination of vascularity using pathology *vs.* angiography. Moreover, we emphasized the importance of confirming hypervascularity before performing embolization.

The accurate selection of patients for embolization is important to minimize complications and unnecessary expenses. Tumor types that are traditionally “hypervascular” may not be hypervascular according to angiographic results. Only patients with tumors angiographically identified as hypervascular exhibited obvious IBL reduction using PE in this study. Further exploration of non-invasive methods to confirm vascularity of tumors before PE is warranted.

## Data Availability Statement

The original contributions presented in the study are included in the article/**Supplementary Material**. Further inquiries can be directed to the corresponding authors.

## Author Contributions

YG: conceptualization, investigation, registration, data curation, visualization, formal analysis, writing—original draft. CW: conceptualization, investigation, methodology, supervision. HL: investigation, registration, data curation, formal analysis, writing—original draft. XL: conceptualization, methodology, project administration, resources, supervision, validation. LJ: conceptualization, funding acquisition, project administration, resources, supervision, validation, writing—review and editing. All authors contributed to the article and approved the submitted version.

## Funding

This study was funded by Peking University Third Hospital (grant number Y71508-01).

## Conflict of Interest

The authors declare that the research was conducted in the absence of any commercial or financial relationships that could be construed as a potential conflict of interest.
