# Supplementary material for: Only Tumors Angiographically Identified as Hypervascular Exhibit Lower Intraoperative Blood Loss Upon Selective Preoperative Embolization of Spinal Metastases: Systematic Review and Meta-Analysis
Source: Front Oncol. 2021 Jan 19;10:597476. doi: 10.3389/fonc.2020.597476 (PMC7874195; doi:10.3389/fonc.2020.597476)
Supplement: Supplementary file 1 [file DataSheet_1.docx]

Sensitivity analysis for intraoperative blood loss (IBL) between embolization group (EG) and non-embolization group (NEG)

# Sensitivity analysis of IBL in all tumor types

## Supplementary Figure 1


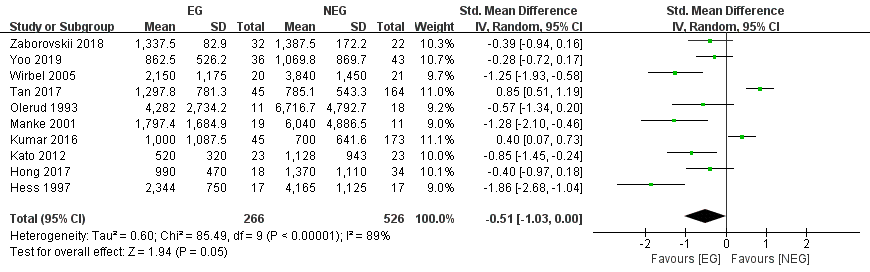


**Supplementary Figure 1.** In the retrospective studies, there was no significant difference between EG and NEG in average IBL (EG: 1395 ml [SD, 1252 ml] vs. NEG: 1402 ml [SD, 1979 ml], P=0.05, SMD=-0.51, 95% CI: -1.03 ~ 0.00, I^2^=89%, random-effect).

## Supplementary Figure 2-3


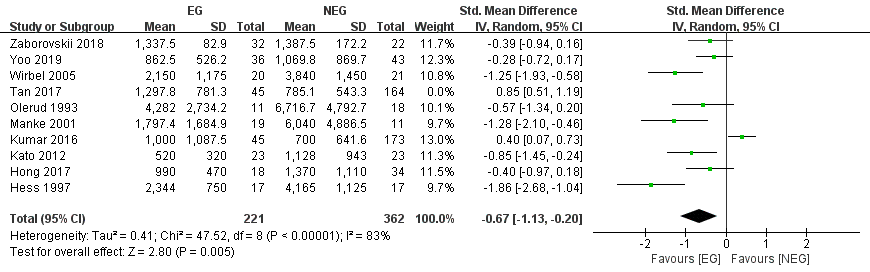

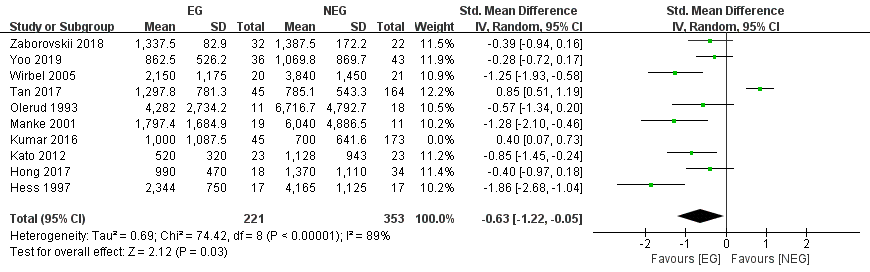


**Supplementary Figure 2-3.** The difference was significant after removing the study of Tan et al. (*P*=0.005, SMD=-0.67, 95% CI: -1.13 ~ -0.20, I^2^=83%, random-effect) or Kumar et al. (*P*=0.03, SMD=-0.63, 95% CI: -1.22 ~ -0.05, I^2^=89%, random-effect).

## Supplementary Figure 4-11


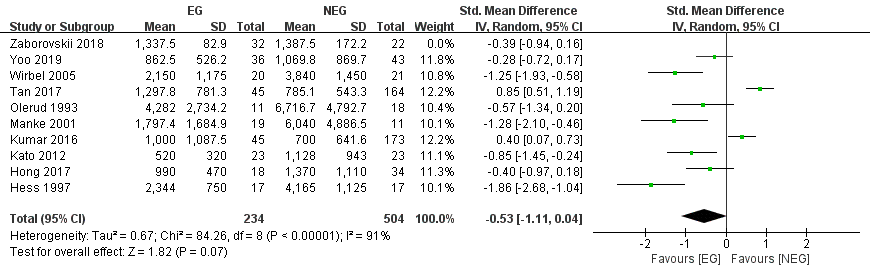

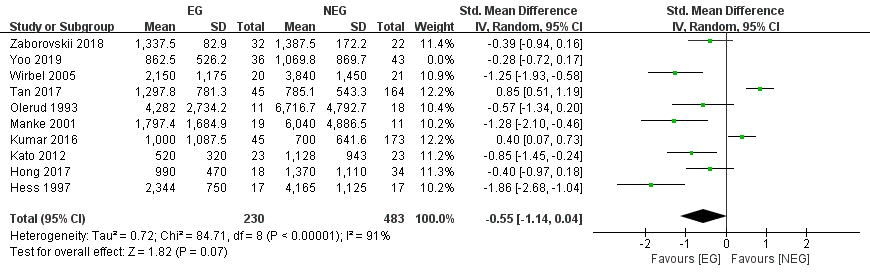

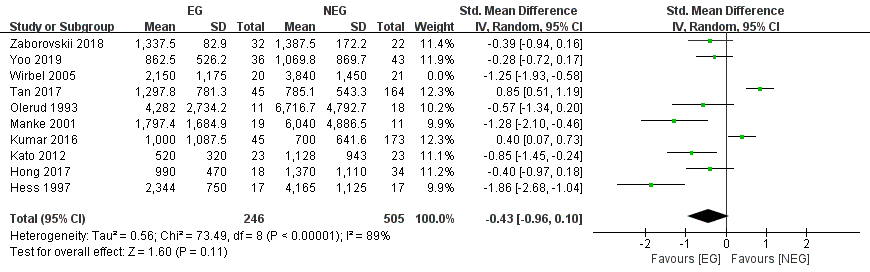

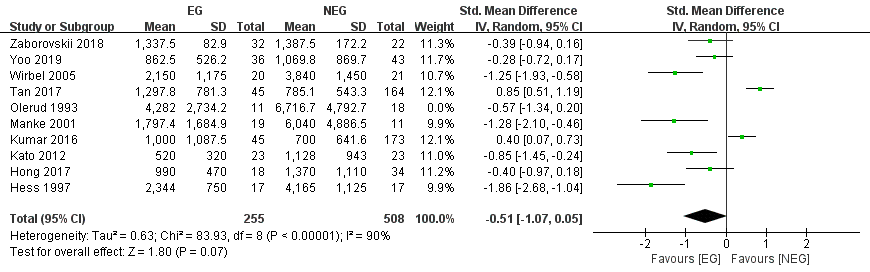

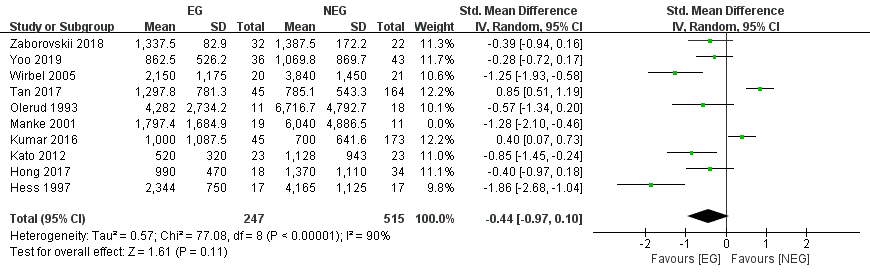

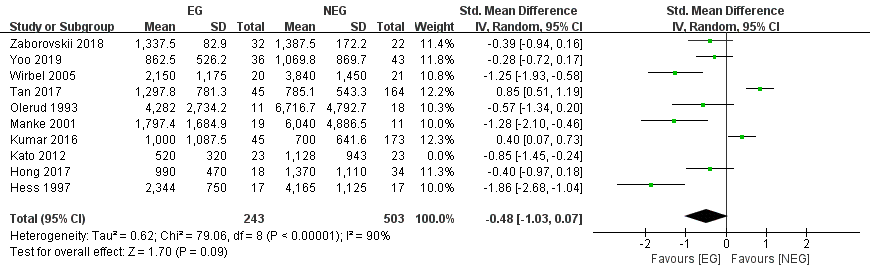

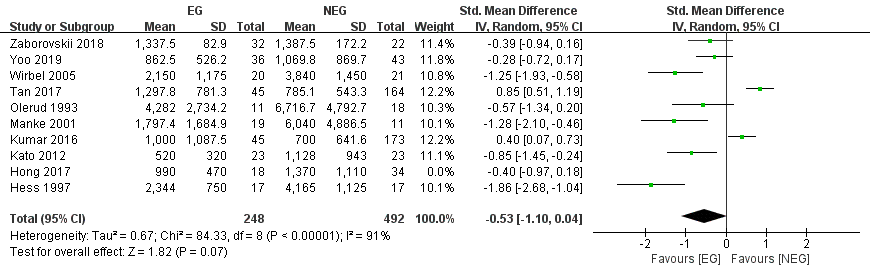

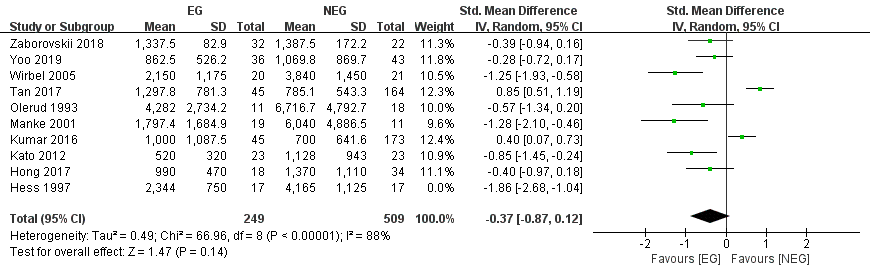


**Supplementary Figure 4-11.** There was no significant difference when omitting other studies.

# Sensitivity analysis of IBL in hypervascular tumors determined as such by consensus

## Supplementary Figure 12


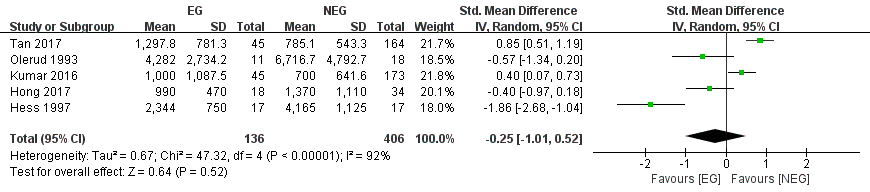


**Supplementary Figure 12.** There was no significant difference between EG and NEG in average IBL (EG: 1531 ml [SD, 1443 ml] vs. NEG: 1202 ml [SD, 1819 ml], P=0.52, SMD=-0.25, 95% CI: -1.01 ~ 0.52, I2=92%, random-effect).

## Supplementary Figure 13


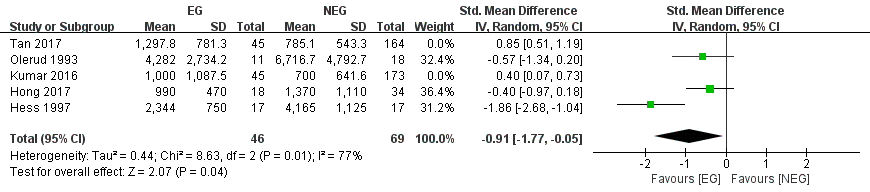


**Supplementary Figure 13.** The difference was significant (P=0.04, SMD=-0.91, 95% CI: -1.77 ~ -0.05, I2=77%, random-effect) only after removing both studies of Tan et al. and Kumar et al.

## Supplementary Figure 14-18


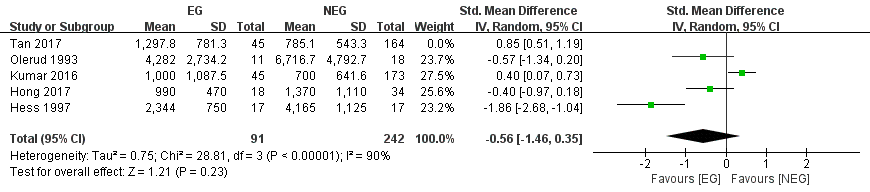

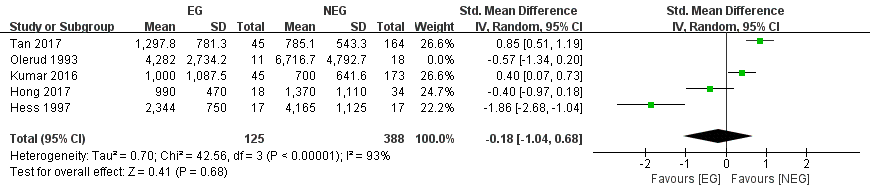

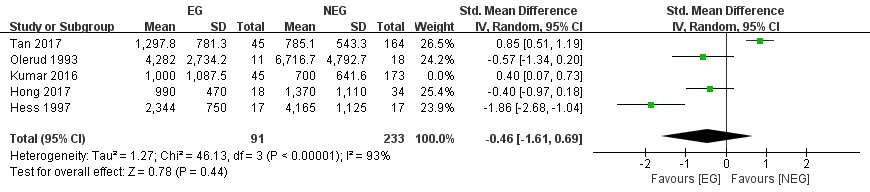

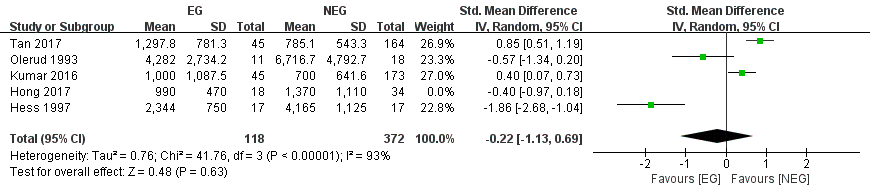

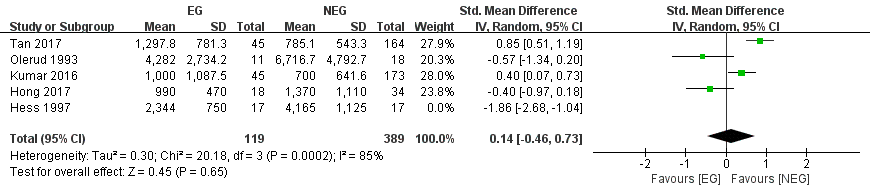


**Supplementary Figure 14-18.** There was no significant difference when omitting other studies.

# Sensitivity analysis of IBL in hypervascular tumors determined as such using angiographic evidence

## Supplementary Figure 19


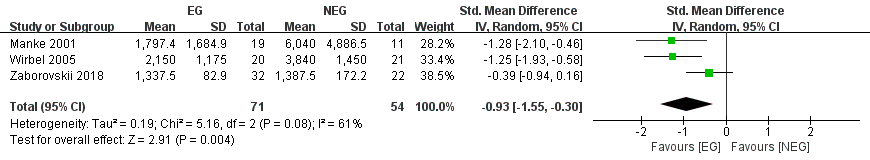


**Supplementary Figure 19.** There was significant difference between EG and NEG in average IBL (EG: 1689 ml [SD, 1108 ml] vs. NEG: 3289 ml [SD, 3042 ml], P=0.004, SMD=-0.93, 95% CI: -1.55 ~ -0.30, I2=61%, random-effect).

## Supplementary Figure 20


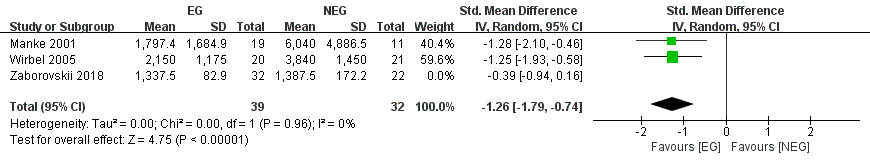


**Supplementary Figure 20.** The difference was significant (P<0.00001, SMD=-1.26, 95% CI: -1.79 ~ -0.74, I2=0%, random-effect) after removing the study of Zaborovskii et al.

## Supplementary Figure 21-22


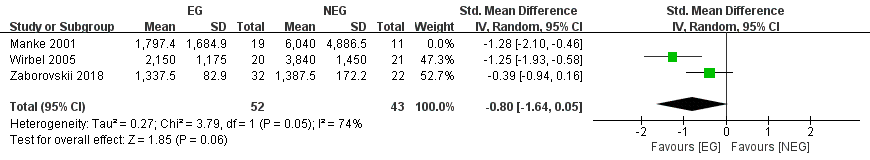

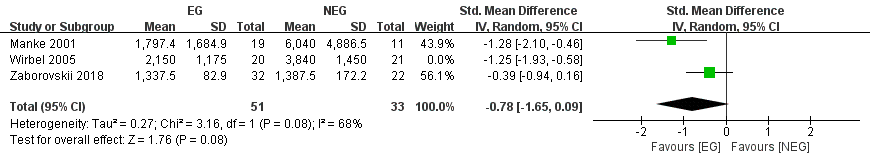


**Supplementary Figure 21-22.** There was no significant difference when omitting other studies.
